# Achieving fertility control through woman’s autonomy and access to maternal healthcare: Are we on track? In-depth analysis of PDHS-2012-13

**DOI:** 10.12669/pjms.316.8354

**Published:** 2015

**Authors:** Sehar-un-Nisa Hassan, Salma Siddiqui, Ayeshah Mahmood

**Affiliations:** 1 Dr. Sehar-un-Nisa Hassan, PhD. Assistant Professor, Department of Behavioral Sciences, S3H, National University of Sciences and Technology (NUST), Islamabad, Pakistan; 2 Dr. Salma Siddiqui, PhD. Assistant Professor & HoD, Department of Behavioral Sciences, S3H, National University of Sciences and Technology (NUST), Islamabad, Pakistan; 3 Ayeshah Mahmood, MPhil. Research Assistant, Department of Behavioral Sciences, S3H, National University of Sciences and Technology (NUST), Islamabad, Pakistan

**Keywords:** Antenatal healthcare, Fertility preferences, Joint decision making, Maternal healthcare, Prenatal healthcare, Women autonomy

## Abstract

**Background and Objective:**

Fertility control preferences and maternal healthcare have recently become a major concern for developing nations with evidence suggesting that low fertility control rates and poor maternal healthcare are among major obstructions in ensuring health and social status for women. Our objective was toanalyze the factors that influence women’s autonomy, access to maternal healthcare, and fertility control preferences in Pakistan.

**Methods:**

Data consisted of 11,761 ever-married women of ages 15-49 years from PDHS, 2012-13. Variables included socio-demographics, women’s autonomy, fertility control preferences and access to maternal healthcare.

**Results:**

Findings from multivariate analysis showed that women’s younger age, having less than three number of children and independent or joint decision-making (indicators of high autonomy) remained the most significant predictors for access to better quality maternal healthcare and better fertility control preferences when other variables were controlled.

**Conclusion:**

Women’s access to good quality maternal health care and fertility control preferences are directly and indirectly influenced by their demographic characteristics and decision-making patterns in domestic affairs.

## INTRODUCTION

Fertility control preferences and maternal healthcare have become a major concern for health professionals, development workers and policy-makers. Existing evidence suggests poor fertility control and maternal healthcare among the major obstructions in ensuring better health and social status of women.^1^ In Pakistan, approximately 30,000 women die every year because of healthcare issues during pregnancy.^2^ Despite this, the rate of progress in this area is slow.^2^,^3^ There is no significant change in fertility preference trends over the years as the recent available statistics show that above than 50% of couples still consider four or more children as ideal family size.^3^ However, women still reported difficulty in accessing professional healthcare (21-35%) and almost 50% reported ‘*no access to professional healthcare facility at the time of delivery*’.^3^ This is a concern as access to better health care is both related to women’s life expectancy and empowerment.

It is important to understand the barriers which result in low access to health care; one way to look at it is the extent to which women exercise their choice in family planning and if it is determined by having more say in other matters as well. It would be interesting to look at the pattern of decision making and mobility, for instance, and whether it explains in any manner both fertility preference and the access to better health care for women.

Literature shows that increased autonomy leads to better access to maternal healthcare,^4^-^6^ at the same time some studies suggest contradictory findings by demonstrating that the rates of independent mobility are very low in women and movement freedom is not a significant predictor for access to maternal healthcare.^7^,^8^ It is important, therefore, to understand patterns of women’s autonomy in context of family system and impact of social and cultural values. It is a prevalent practice in Pakistani society that women when step out of house are accompanied by either a male family member or another woman. This is due to both a safety measure against untoward harassment and also to keep the image of a “good woman”. Moreover, in household matters women rarely make decisions independently^3^ a common phenomenon in a collectivistic culture, with some positive outcomes on access to maternal healthcare and fertility control preferences.^7^,^9^

The joint decision-making in household matters and accompanied movement does not mean absence of autonomy in the context of Pakistani society and therefore this study takes both individual and joint decision-making as indicator of high autonomy in contrast to conditions where household decisions are solely taken by husbands or other family members. This study investigates the pathway that explains the access to maternal healthcare and fertility preferences for Pakistani women and whether their decision making along with selected other demographic variables helps determine it.

**Fig.1:**
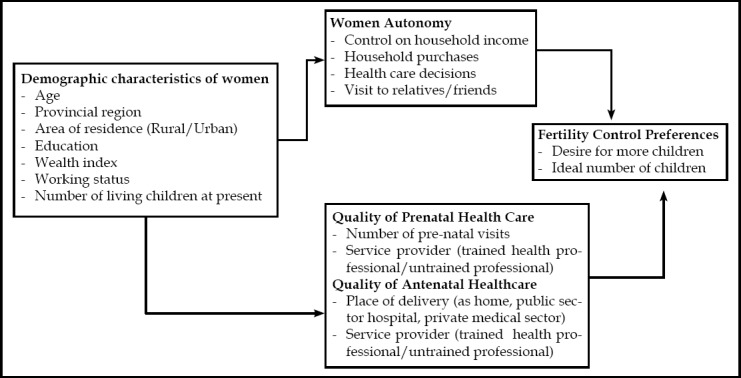
Conceptual Model.

## METHODS

The data was obtained using stratified sampling. A two-stage stratified sampling technique was used in PDHS-2012-13 survey. In the first stage, 248 urban areas and 252 rural areas were chosen by employing a probability proportional to size sampling scheme with an independent selection in each sampling stratum. In the second stage, a systematic, sampling technique was used to select households from each sample point with a random start. A total of 14,000 households were chosen for data collection.

Further details of data collection may be taken from Pakistan Demographic Health Survey (PDHS) 2012–2013.^3^ The data used in the analysis consisted of (N=11761) ever-married women. Questionnaire used was ‘*Woman Household Questionnaire’*, developed by Demographic Health Surveys.

### Variables

### Socio-demographic Characteristics

It included; age, region, residence (urban and rural), education, wealth index, working status and number of living children.

### Autonomy

It consisted of women’s independent or joint control on income, purchases, healthcare decisions, and visits to relatives.^3^ Total score was obtained by summating autonomy indicators (3=independent, 2=joint, 1=husband, 0=others). Higher scores were indicative of high autonomy and low scores were indicative of low autonomy.

### Women’s maternal healthcare

**Fig.2:**
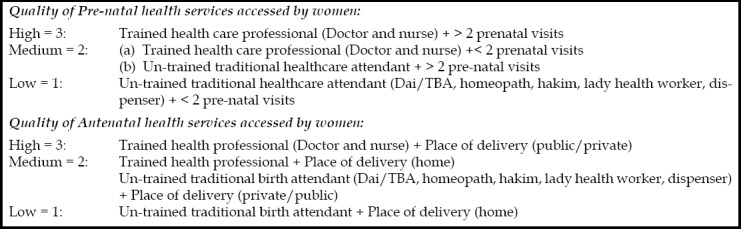
Indicators to determine quality of maternal health care.

Women who gave birth in the last five years were inquired about receiving maternal healthcare for most recent birth, including number of visits, service provider and place of healthcare. It was measured as quality of prenatal and antenatal healthcare.^3^ Indicators used to determine quality of maternal healthcare are shown in Fig.2.

### Fertility Control Preferences

The fertility control preference of women were assessed through (1) desire for more children^3^ and (2) ideal number of children.^3^

### Statistical Analysis

Analysis was done using SPSS software. Mean scores, standard deviations, ANOVA and t-test were applied to determine associations among variables with p-value less than 0.05. Moderation analysis was carried out to determine the interaction effects among the variables.

## RESULTS

Findings showed that rates of independent autonomy are very low (8-11%) in this nationally representative sample of women from Pakistan. Similarly less number of women (10%) reported access to good quality maternal health care, 58% reported access to medium quality healthcare and 32% were getting low quality care. 64% of couples considered four or more children as ideal family size and 23% of women and 30% of men wanted to have another child within two years of previous childbirth. Table-I & II.

**Table-I:** Mean differences on women autonomy, access to maternal healthcare and fertility preferences across demographics (N = 11761).

Variable	Categories	High autonomy	Low autonomy	Prenatal	Antenatal	Desire	Ideal number
		M(SD)	M(SD)	M(SD)	M(SD)	M(SD)	M(SD)
Age	15 – 19	.27(.44)	.73(.44)	1.87(.75)	1.68(.57)	5.75(.69)	3.93(1.35)
	20 – 24	.35(.48)	.65(.48)	1.64(.75)	1.63(.59)	5.51(.90)	3.92(1.33)
	25 – 29	.43(.50)	.57(.50)	1.58(.72)	1.61(.58)	5.10(1.05)	4.12(1.34)
	30 – 34	.52(.50)	.48(.50)	1.57(.71)	1.58(.58)	4.73(1.04)	4.32(1.39)
	35 – 39	.55(.50)	.45(.50)	1.63(.75)	1.54(.56)	4.40(.93)	4.59(1.43)
	40 – 44	.58(.49)	.42(.49)	1.62(.78)	1.48(.61)	4.15(.77)	4.90(1.41)
	45 – 49	.49(.50)	.51(.50)	1.52(.76)	1.40(.53)	4.11(.73)	5.35(1.20)
	F			10.02***	13.61***	374.71***	93.68***
Region	Punjab	.61(.49)	.39(.49)	1.67(.76)	1.61(.58)	4.85(1.07)	3.77(1.26)
	Sindh	.50(.50)	.50(.50)	1.66(.72)	1.65(.52)	5.10(1.07)	4.40(1.33)
	KPK	.36(.48)	.64(.48)	1.62(.76)	1.65(.66)	4.94(.99)	4.21(1.41)
	Baluchistan	.27(.44)	.73(.44)	1.39(.70)	1.27(.49)	4.90(1.13)	5.20(1.20)
	Gilgit Baltistan	.38(.48)	.62(.48)	1.60(.72)	1.57(.55)	4.98(1.07)	4.41(1.25)
	Islamabad	.56(.50)	.44(.50)	1.68(.58)	1.91(.42)	4.81(1.06)	3.35(1.38)
	F			44.07***	184.82***	18.70***	370.96***
Residence	Urban	.49(.50)	.51(.50)	1.66(.68)	1.72(.53)	4.90(1.09)	4.01(1.44)
	Rural	.44(.50)	.56(.50)	1.57(.77)	1.49(.60)	4.97(1.05)	4.44(1.34)
	T			7.17***	20.91***	3.33***	16.63***
Education	Illiterate	.41(.49)	.59(.49)	1.52(.77)	1.43(.58)	4.90(1.07)	4.70(1.32)
	Primary	.50(.50)	.50(.50)	1.68(.75)	1.68(.57)	4.90(1.07)	3.94(1.29)
	Secondary	.47(.50)	.53(.50)	1.73(.66)	1.82(.50)	5.04(1.07)	3.63(1.26)
	Higher	.63(.48)	.37(.48)	1.75(.56)	1.94(.33)	5.03(1.04)	3.36(1.22)
	F			70.66***	514.88***	12.94***	661.11***
Wealth Index	Poorest	.39(.49)	.61(.49)	1.44(.76)	1.30(.52)	5.01(1.03)	4.96(1.22)
	Poorer	.46(.50)	.54(.50)	1.56(.78)	1.49(.61)	4.96(1.08)	4.49(1.33)
	Middle	.48(.50)	.52(.50)	1.65(.78)	1.59(.60)	4.87(1.08)	4.19(1.32)
	Richer	.49(.50)	.51(.50)	1.70(.71)	1.75(.56)	4.89(1.06)	3.85(1.37)
	Richest	.48(.50)	.52(.50)	1.72(.58)	1.90(.39)	4.95(1.10)	3.58(1.32)
	F			62.69***	445.42***	7.25***	415.84***
Working	No	.41(.49)	.59(.49)	1.61(.73)	1.61(.58)	4.96(1.07)	4.22(1.40)
	Yes	.66(.47)	.34(.47)	1.59(.76)	1.47(.56)	4.84(1.05)	4.36(1.38)
	t			1.28	10.50***	4.59***	4.09***
No. of children	≤ 3 children	.28(.45)	.72(.45)	1.67(.72)	1.68(.56)	5.37(.96)	3.85(1.31)
	>3 children	.42(.49)	.58(.49)	1.53(.75)	1.46(.59)	4.36(.93)	4.78(1.34)
	t	.51(.50)	.49(.50)	11.65***	10.31***	1716.32***	715.75***
Autonomy	Low autonomy			1.58(.74)	1.56(.59)	5.11(1.04)	4.44(1.40)
	High autonomy			1.64(.73)	1.62(.57)	4.74(1.07)	4.03(1.37)
	t			5.02***	6.00***	19.21***	16.01***
Prenatal healthcare quality	High					2.06 (1.07)	4.29 (1.36)
	Medium					1.97 (1.07)	3.76 (1.32)
	Low					2.17 (1.09)	4.81 (1.33)
	F					19.86***	298.36***
Antenatal healthcare quality	High					2.00 (1.01)	4.19 (1.37)
	Medium					2.01 (1.08)	3.85 (1.34)
	Low					2.13 (1.06)	4.69 (1.33)
	F					18.97***	362.32***

Note. High autonomy = Women independent and joint, Low autonomy = husband alone and others. Desire = (2= wanted another child, 1=undecided and 0= no more); Ideal number = (0= no children, 1 =1 - 2 children, 2 = 3 – 4 children, and 3=5 or more),

*** p<.001

**Table-II:** Interaction effect among variables selected for analysis (N = 11761).

	Prenatal					Antenatal				
Variable	M	SD	∆R^2^	Β	F	M	SD	∆R^2^	Β	F
Desire for more children													
Age	3.52	1.26	.16	-.40	2182.34***	3.52	1.26	.16	-.40	2182.34***
Region	2.76	1.53	.00	.01	1091.44***	2.76	1.53	.00	.01	1091.44***
Education	.82	1.07	.00	.02	551.09***	.82	1.07	.00	.02	551.09***
Wealth	2.88	1.43	.00	-.08	450.10***	2.88	1.43	.00	-.08	450.10***
No. of Children	1.43	.51	.10	-.40	699.79***	1.43	.51	.10	-.40	699.79***
Work status	.19	.39	.00	-.01	599.91***	.19	.39	.00	-.01	599.91***
Autonomy	1.49	.50	.03	-.10	280.61***	1.46	.50	.03	-.27	369.44***
Prenatal healthcare	1.72	.93	.00	-.41	151.78***	1.59	.58	.00	-.59	210.05***
Age*Autonomy*Prenatal	9.18	7.54	.10	.08	444.75***	8.32	5.63	.10	.10	607.60***
Region*Autonomy*Prenatal	6.74	6.16	.00	.01	102.81***	6.27	5.25	.00	.01	142.91***
Education*Autonomy*Prenatal	2.46	3.84	.01	-.02	119.79***	2.34	3.58	.00	-.02	150.82***
Wealth*Autonomy*Prenatal	7.80	6.71	.00	.00	101.79***	7.27	5.85	.00	.01	143.65***
No. of children*Autonomy*Prenatal	8.67	8.88	.18	.07	779.67***	8.16	6.67	.19	.09	1141.81***
Work status*Autonomy*Prenatal	.54	1.40	.00	.01	102.32***	.46	1.11	.00	.06	44.01***
Ideal number of children													
Age	3.51	1.26	.04	.21	537.08***	3.51	1.26	.04	.21	537.08***
Region	2.75	1.53	.01	.11	349.89***	2.75	1.53	.01	.11	349.89***
Education	.82	1.07	.10	-.35	678.54***	.82	1.07	.10	-.35	678.54***
Wealth	2.88	1.43	.02	-.21	626.57***	2.88	1.43	.02	-.21	626.57***
No. of Children	1.43	.51	.02	.19	594.81***	1.43	.51	.02	.19	594.81***
Work status	.19	.39	.00	-.03	511.68***	.19	.39	.00	-.03	511.68***
Autonomy	1.48	.50	.02	-.76	134.17***	1.46	.50	.02	-.85	256.52***
Antenatal healthcare	1.72	.93	.00	-.38	80.45***	1.59	.58	.06	-.93	511.13***
Age*Autonomy*Antenatal	6.14	7.52	.03	.06	148.12***	8.29	5.62	.03	.07	487.50***
Region*Autonomy*Antenatal	6.72	6.15	.00	.01	58.12***	6.25	5.24	.00	.02	351.63***
Education*Autonomy*Antenatal	2.46	3.83	.08	-.11	310.57***	2.34	3.57	.06	-.11	625.56***
Wealth*Autonomy*Antenatal	7.78	6.69	.06	-.08	252.03***	7.25	5.84	.05	-.08	572.78***
No. of children*Autonomy*Antenatal	8.64	8.87	.09	.06	360.15***	8.14	6.67	.09	.08	825.28***
Work status*Autonomy*Antenatal	.54	1.39	.00	.06	62.26***	.46	1.11	.00	-.02	3.61

Note.

*** p<.001

## DISCUSSION

The main objectives of this in-depth analysis was to determine the nature of inter-relationship between women’s demographic characteristics, autonomy, access to maternal health care and fertility control preferences. Findings revealed that 45% of women who were categorized as having “High Autonomy” also more likely to report access to good quality maternal health care as well as better fertility control preferences as compared to other women. As “High autonomy” in this analysis was determined by combining response by women who reported individual decision making and joint decision making in areas of income control, household purchase, healthcare decision and travel, it is important to understand the position of women in family system as well role of social and cultural values to understand the findings. Traditionally, women are not encouraged to take independent decisions even in household matters until their old-age. It is a rare phenomenon that women take independent decisions related to their own healthcare and fertility control.^10^,^11^ Also in the context of post-natal healthcare it is more likely that women depend on their care-takers at the time of delivery or in conditions of ill-health. Thus women’s solo-decision as a pathway to access high quality maternal health care and positive fertility control outcomes is not achievable on realistic ground. Analysis also showed that women in younger age, with less than three children and employed status were more involved in joint decision-making and reported access to better quality maternal health care. This is consistent with findings from another study^12^ where in case of young couples husband involvement was found to be a significant predictor for an access to good quality reproductive healthcare at the time of delivery.

Women’s fertility preferences were the main outcome variable for this analysis. Consistent with other evidence^13^,^14^ the present study findings revealed that overall women in younger age groups, with higher education, better economic conditions, urban residence, and currently having less than three children had better fertility preferences. A case-study from Punjab^1^ and studies from other developing countries^5^,^15^,^16^ have confirmed this pattern of relationship. The positive influence of women’s education with fertility control has also been indicated by study from Bangaladesh.^17^

The main purpose of this study was to determine whether women’s autonomous status and access to maternal health care will independently or after interaction predict women’s fertility control preferences. Findings from multivariate analysis showed that women’s younger age, having less than three number of children and independent or joint decision-making (indicators of high autonomy) remained the most significant predictors for access to better quality maternal healthcare and better fertility control preferences when other variables were controlled. Findings also revealed that quality of ante-natal healthcare accessed by women significantly influence women’s choice for ideal number of children independently and even after interaction with other variables.

### Recommendations

Findings strongly suggest that joint decision-making in couples maximize access to better quality ante-natal and post-natal healthcare which lead to positive outcomes in fertility control. To achieve success in fertility control, programs should target young, less educated women living in rural areas of Sindh, Balochistan and Giglit Baltistan. It is recommended to conduct further analysis by including other variables from PDHS 2012-13 data such as media exposure, contraceptive use patterns, and experience of domestic abuse and women’s and men’s attitudes towards wife beating which may explain role of other factors in determining women’s fertility preferences.
